# Supplementary material for: Evaluating pictorial support in person-centred care for children (PicPecc): a protocol for a crossover design study
Source: BMJ Open. 2021 May 4;11(5):e042726. doi: 10.1136/bmjopen-2020-042726 (PMC8098982; doi:10.1136/bmjopen-2020-042726)
Supplement: Supplementary data [file bmjopen-2020-042726supp001.pdf]

Supplementary file 1. Interview questions in the process evaluation

Main questions to the child:

Tell me about your thoughts about getting your treatment.

Did the healthcare professionals listen to your wishes?

Tell me about a situation when you got support.

Main questions to legal guardians:

Tell me about your child's care.

Did the healthcare professionals listen to you and your child´s wishes?

Tell me about a situation when your child got support.

Main questions to healthcare professionals:

Tell me about your experience of caring for the child.

Do you think that the child felt listened to?

Tell me about a situation when the child got support.
